# Fate of turbid glacial inflows in a hydroelectric reservoir

**DOI:** 10.1007/s10652-021-09815-4

**Published:** 2021-09-26

**Authors:** Daniel M. Robb, Roger Pieters, Gregory A. Lawrence

**Affiliations:** 1 Department of Civil Engineering, University of British Columbia, Vancouver, BC Canada; 2 Department of Earth, Ocean and Atmospheric Sciences, University of British Columbia, Vancouver, BC Canada

**Keywords:** Physical limnology, Horizontal dispersion, Turbidity, Glacial inflow, Particle settling, Light attenuation

## Abstract

Turbidity from glacial meltwater limits light penetration with potential ecological consequences. Using profiles of temperature, conductivity, and turbidity, we examine the physical processes driving changes in the epilimnetic turbidity of Carpenter Reservoir, a long and narrow, glacier-fed reservoir in southwest British Columbia, Canada. Following the onset of permanent summer stratification, the relatively dense inflows plunged into the hypolimnion, and despite the high glacial load entering the reservoir, the epilimnion cleared due to particle settling. Using a one-dimensional (longitudinal) diffusion equation for a decaying substance to describe the variation in epilimnetic turbidity, we obtain two nondimensional parameters: the epilimnetic inflow parameter, $\mathcal {I}$, a measure of the turbidity flux into the epilimnion; and the dispersion parameter, ${\mathcal {D}}$, a measure of longitudinal dispersion. In the case of Carpenter Reservoir: $\mathcal {I}\ll 1$, indicating that turbidity declines over the summer; and ${\mathcal {D}}\ll 1$, indicating a strong gradient in turbidity along the epilimnion. Using our theoretical formulation of epilimnetic turbidity variations in conjunction with monthly field surveys, we compute the particle settling velocity (${\sim}{0.25}\,{\hbox {m}\,\hbox {d}^{-1}}$), the longitudinal dispersion coefficient (50–70 ${\hbox {m}^{2}\,\hbox {s}^{-1}}$), and the flux of turbid water into the epilimnion (${\sim }1{\%}$ of the total inflow). Our approach is applicable to other reservoirs and can be used to investigate changes in turbidity in response to changes in $\mathcal {I}$ and ${\mathcal {D}}$.

## Introduction

Inflows of glacial meltwater into lakes and reservoirs are typically colder [30], and more turbid [38] than non-glacial inflows. The magnitude of glacial and non-glacial inflows varies during spring and summer. During freshet snowmelt dominates, while later in the summer, when the snow has melted and non-glacial inflows are declining, glacial inflows augment streamflow [31] and elevate turbidity [19]. Climate-driven changes in glacial coverage also influence glacial discharge [44] and turbidity [36].

The temperature and turbidity of glacial inflows affect the depths to which they plunge [1], and the thermal stratification of the receiving water body [4]. Turbidity from glacial meltwater can control the attenuation of sunlight into the water column, affecting the distribution of heat [16], the level of biological activity [12], and the aesthetics of the surface water [41]. Anthropogenic change, such as damming for hydropower projects, can lead to particle retention behind dams [49], and to a shifting of the seasonal particle flux from summer to winter [11].

**Fig. 1:**
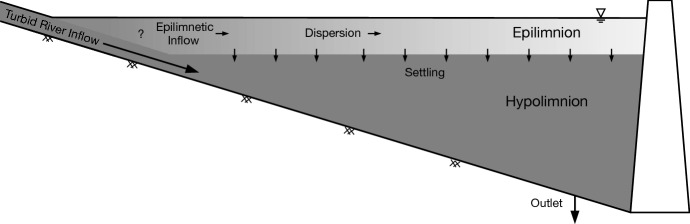
Schematic showing a glacially-turbid river inflow entering a reservoir with a deep outlet. The inflow is denser than the epilimnion and plunges into the hypolimnion. The epilimnetic inflow is a small fraction of the river inflow that makes its way into the epilimnion at the upstream end of the reservoir. Turbidity is dispersed along the epilimnion and declines as suspended particles settle out of the epilimnion. These processes lead to a longitudinal gradient in turbidity along the epilimnion, as indicated by the grey shading. The question mark represents processes at the upstream end of the epilimnion that lead to the epilimnetic inflow. A detailed understanding of these processes is unnecessary for the present study

During summer, a two-layer stratification develops in most lakes, with a warmer, less dense surface layer (epilimnion) overlying a cooler, denser layer (hypolimnion, Fig. 1). In natural lakes, outflows are typically from the water surface, so before water leaves the lake it passes through the epilimnion where light intensity is high, and nutrients can be used for biological productivity. In contrast, deep outlets in reservoirs can allow inflows to bypass the epilimnion: denser inflows can plunge below the epilimnion, passing through the hypolimnion to the deep outlets, making inflowing nutrients unavailable for productivity [5, 27, 35]. We examine a case in which turbid inflows from glacial meltwater pass through a hydroelectric reservoir with deep outlets. Of particular interest is the effect of these inflows on the light regime in the reservoir.

Inflows of glacial meltwater can alter the light climate in a receiving water body by reducing light penetration into the water column. While dissolved substances (e.g. humic acids) and organic particles (e.g. phytoplankton) control light attenuation in many temperate lakes [22], inorganic glacial particles can play a dominant role in attenuating light in glacier-fed lakes [14]. In a study of 18 glacier-fed lakes with widely varying turbidity, glacial particles accounted for about two thirds of light attenuation across all lakes, rising to 90% for lakes with high turbidity ($>20$ NTU, [38]). Since sunlight is a fundamental component of many physical, chemical, and biological processes, understanding the effect of glacially-turbid inflows on the light regime in a reservoir is needed for understanding the ecological function of the reservoir [2, 25, 43] and for informing reservoir management decisions [3, 15, 21].

Here we examine Carpenter Reservoir, part of the Bridge River Hydroelectric Project, located in southwest British Columbia, Canada. We will show that thermal stratification in summer almost completely isolates the epilimnion from glacial inflows, and, instead, these inflows pass through the hypolimnion to the deep outlets. During summer, glacial fines settle from the epilimnion and the turbidity in the epilimnion declines. The relatively clear epilimnion during summer was unexpected given the high glacial inflow during this period. This phenomenon may occur in other glacier-fed reservoirs in similar settings.

The goal of this paper is to investigate the interactions of glacial inflows, wind, and stratification and their effect on turbidity and light attenuation in the epilimnion of a hydroelectric reservoir. We examine data from a two-year field study of Carpenter Reservoir, collected from spring to fall in 2015 and 2016. A description of the study area and field methods is provided in Sect. 2. The evolution of temperature, conductivity, and turbidity during the study period is described in Sect. 3. Then, in Sect. 4, we present a theoretical analysis of epilimnetic turbidity variation that incorporates longitudinal dispersion and particle settling. We apply this theory to Carpenter Reservoir and discuss the implications in Sect. 5, followed by conclusions in Sect. 6.

## Methods

### Study site

**Fig. 2:**
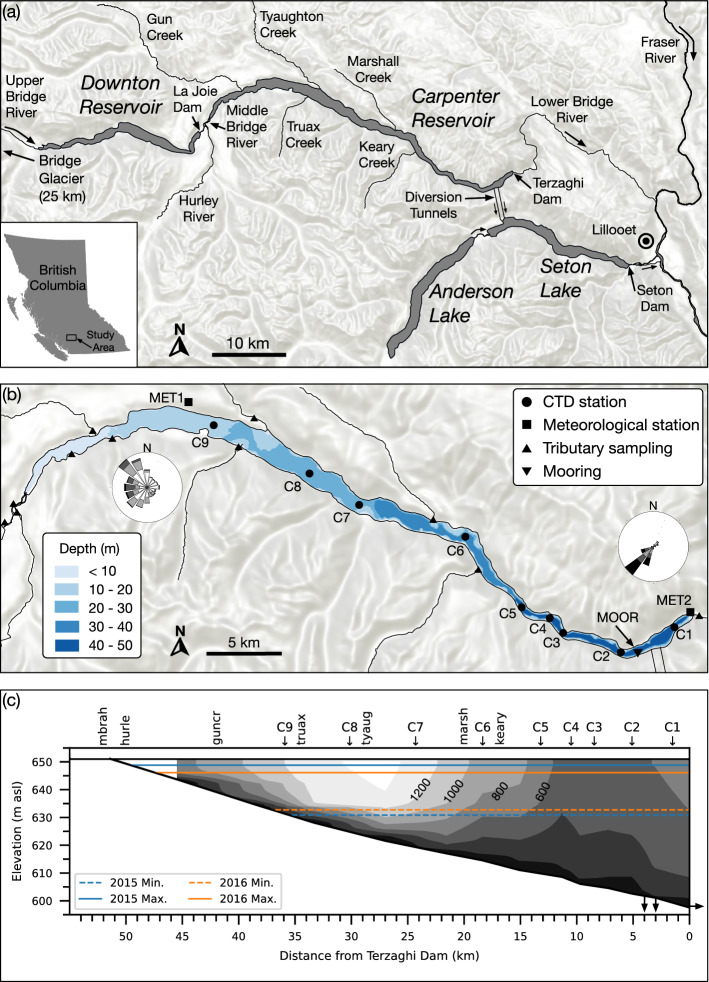
**a** Map of the study area. **b** Plan view of Carpenter Reservoir and monitoring stations. The contours indicate the depth of water below full pool (651.08 m asl). Terrain background image: © Mapbox. **c** Profile view of Carpenter Reservoir showing the minimum and maximum water level in 2015 and 2016, and showing contours of reservoir width at 200-m intervals from $200\,{\rm m}$ to $1400\,{\rm m}$. CTD stations are marked C1–C9; tributary sampling stations are Middle Bridge above Hurley River (mbrah), Hurley River (hurle), Gun Creek (guncr), Truax Creek (truax), Tyaughton Creek (tyaug), Marshall Creek (marsh), and Keary Creek (keary). The downward arrows at the reservoir bottom mark the twin tunnels to Seton Lake. The rightward arrow at the dam marks the outflow to the Lower Bridge River

Carpenter Reservoir $(50^\circ 51^\prime \,\mathrm {N},\; 122^\circ 30^\prime \,\mathrm {W})$ is part of the Bridge River Hydroelectric Project, located 200 km north of Vancouver, British Columbia, Canada (Fig. 2a). The reservoir lies on the original floodplain of the Bridge River, and was formed by the construction of Terzaghi Dam in 1960. The reservoir is long (${\sim}{50}\,{\hbox {km}}$) and narrow (${\sim}{1}\,{\hbox {km}}$), with a maximum operating level of ${651.08}\,{\hbox {m}\,\hbox {asl}}$, a maximum depth of ${50}\,\hbox {m}$, a maximum surface area of ${46}\,{\hbox {km}}^{2}$, and a maximum volume of $1.0\times 10^9\,{\hbox {m}^3}$. The reservoir has steep valley walls on both sides with mountain peaks reaching nearly ${3000}\,{\hbox {m}\,\hbox {asl}}$.

The Bridge Glacier is located at the headwaters of the Bridge River. Meltwater is high in glacial fines which are slow to settle, giving the water a characteristic cloudy (turbid) appearance. Water from the Upper Bridge River first flows into Downton Reservoir created by La Joie Dam. Leaving La Joie Dam is the Middle Bridge River, which is the largest inflow (27%) to Carpenter Reservoir. Other inflows to Carpenter Reservoir include Tyaughton Creek (20%), the Hurley River (18%), and Gun Creek (16%), all located in the upper reaches of the reservoir. From Carpenter Reservoir most of the water is diverted through twin tunnels to Seton Lake Reservoir (${236.36}\,{\hbox {m}\,\hbox {asl}}$ at full pool) with an elevation drop of ${\sim}{400}\,{\hbox {m}}$ used to generate hydroelectricity, while a smaller amount is passed through low-level outlets at Terzaghi Dam to maintain minimum flows in the Lower Bridge River (Fig. 2a). These outlets are all from the deepest part of the reservoir (Fig. 2c).

In Carpenter Reservoir nutrient levels were low during the study period, with soluble reactive phosphorus and total dissolved phosphorus averaging $1.2\,{\upmu \hbox {g}\,\hbox {L}^{-1}}$ and $2.3\,{\upmu \hbox {g}\,\hbox {L}^{-1}}$ (near the detection limits of $1.0\,{\upmu \hbox {g}\,\hbox {L}^{-1}}$ and $2.0\,{\upmu \hbox {g}\,\hbox {L}^{-1}}$, respectively) and nitrate averaging ${10.3}\,{\upmu \hbox {g}\,\hbox {L}^{-1}}$ (detection ${5}\,{\upmu \hbox {g}\,\hbox {L}^{-1}}$). Oxygen concentrations were generally high, $\ge 8\,{\hbox {mg}\,\hbox {L}^{-1}}$, and close to saturation, $\ge 80{\%}$, and chlorophyll concentrations were generally low, $<2\,{\upmu \hbox {g}\,\hbox {L}^{-1}}$, all of which are consistent with a phosphorus-limited oligotrophic system [50]. Resident fish species include kokanee (*Oncorhynchus nerka*), a landlocked sockeye salmon, as well as sport fish such as bull trout (*Salvelinus confluentus*), rainbow trout (*Oncorhynchus mykiss*), and mountain whitefish (*Prosopium williamsoni*). Terzaghi Dam blocks the passage upstream of anadromous salmon and steelhead.

### Data collection

Field data were collected from spring to fall in 2015 and 2016. Measurements included profiles of water column properties, tributary sampling, a temperature mooring, and meteorological data.

#### Profiles

Monthly CTD (conductivity, temperature, depth) profiles were collected using a Sea-Bird SBE 19plus V2 (accuracy ${\pm 0.005}\,^\circ \hbox {C}$, ${\pm 1}\,{\upmu \hbox {S}\,\hbox {cm}^{-1}}$) from 22 May to 20 October 2015 and 13 April to 14 October 2016. Measured conductivity was converted to conductivity at ${25}\,^\circ \hbox {C}$, $C_{\mathrm {25}}$, following Pawlowicz [34]. The CTD was equipped with a WETLabs ECO combined fluorometer and optical backscatter (turbidity) sensor, a Biospherical photosynthetically active radiation (PAR) sensor, and a SBE 43 dissolved oxygen sensor. Profiles were collected monthly at up to nine locations (C1–C9, Fig. 2b). Turbidity from the CTD was calibrated to bottle data.

#### Tributary sampling

Water temperature was measured at 20-min intervals in major tributaries using Onset Hobo TidbiT v2 temperature loggers (UTBI-001, accuracy ${\pm 0.25}\,^\circ \hbox {C}$). From the same tributaries, conductivity and turbidity were measured monthly using a YSI multi-parameter probe. Data from the YSI were calibrated to bottle data. The tributary sampling covered 89% of the total drainage into Carpenter Reservoir.

#### Temperature mooring

Time-series measurements of water temperature were obtained from temperature loggers attached to a taut-line mooring hanging from a log boom. The mooring was located upstream of the twin diversion tunnels at the location of greatest cross-channel depth (Fig. 2b). The mooring was deployed from 16 April to 20 October 2015, and 13 April to 14 October 2016. In 2015, temperature was recorded at 11 depths: 0.5, 1, 2, 3, 5, 7, 10, 15, 20, 25 and 30 m. In 2016, additional temperature sensors were added at 8 depths: 8, 9, 11, 12, 13, 14, 16 and 18 m to better resolve the thermocline. In 2016, four sensors were also moored at 0.3, 1.7, 7 and ${12}\,{\hbox {m}}$ above the reservoir bottom, approximately ${1}\,{\hbox {km}}$ downstream of the log boom. The loggers were mostly the Onset Hobo Water Temperature Pro v2 (U22-001, accuracy ${\pm 0.2}\,^\circ \hbox {C}$, 20-min intervals) as well as several of the RBR Solo T (accuracy ${\pm 0.002}\,^\circ \hbox {C}$, 10-s intervals) at selected depths.

#### Meteorological data

Meteorological data were collected at two locations (Fig. 2b). At Terzaghi Dam, wind speed and direction data were collected by BC Hydro using an RM Young 05103 wind monitor at approximately ${5}\,{\hbox {m}}$ above the crest of the dam. A second station was installed at the same location, consisting of an Onset Hobo Micro Station (H21-002) data logger with PAR (S-LIA) and solar radiation (S-LIB) sensors. Relative humidity and air temperature were measured with an Onset Hobo Pro (U23-001). At Five Mile Station, located near the upstream end of the reservoir, wind speed and direction data were collected by BC Wildfire Service.

### Light attenuation and turbidity

**Fig. 3:**
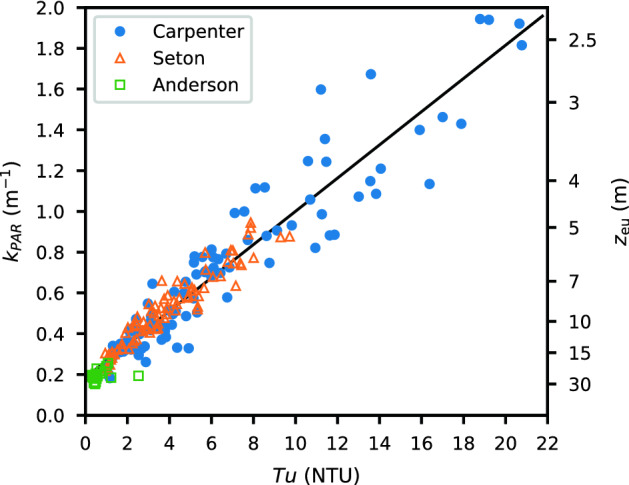
Scatter plot of light attenuation coefficient $k_{\mathrm {PAR}}$ versus turbidity $Tu$. Each data point corresponds to one CTD profile. The turbidity shown is the depth-averaged value from just below the water surface to the euphotic depth $z_{\mathrm {eu}}$. The light attenuation coefficient was calculated as $k_{\mathrm {PAR}} = \ln {100}/z_{\mathrm {eu}}$. Measurements from Carpenter Reservoir, Seton Lake and Anderson Lake are included in the regression. The linear least squares fit is $k_{\mathrm {PAR}} = 0.081 Tu + 0.185$ ($R^2 = 0.9$, $N = 206$)

In Carpenter Reservoir, as in many glacier-fed water bodies, light attenuation is dominated by glacial fines. The standard measure of suspended particulates is the method of total suspended solids; however, this method is both time consuming and inaccurate for fine particles. Instead, in this system, we use turbidity and find that it can quantitatively predict the light attenuation, the key parameter of interest. To evaluate the relationship between turbidity and light attenuation, we use data from all the profiles in Carpenter Reservoir, as well as those from adjacent Seton and Anderson Lakes [26]. The light attenuation coefficient, $k_{\mathrm {PAR}}$, was calculated by fitting an exponential decay to the PAR profile from just below the water surface to the euphotic depth (the depth where light intensity was 1% of that just below the water surface). The light attenuation coefficient is compared to the depth-averaged turbidity in the euphotic zone in Fig. 3 and was found to be well-correlated $(R^2 = 0.9)$. As a result, we use turbidity as a proxy to characterize the light regime in Carpenter Reservoir. Similar close relationships between turbidity and light attenuation have been reported for other glacier-fed water bodies including Coquitlam Reservoir and Harrison Lake in British Columbia (unpublished), 23 turbid lakes in Alaska [23, Table 4], and 18 lakes in Chile, New Zealand, and the Rocky Mountains [38, Table 2].

### Interfacial displacement due to wind forcing

To determine the effect of wind forcing on basin-scale internal motion, we compute the Wedderburn number, $W = \frac{g^{\prime }{h_{1}}^{2}}{u_{*}^{2}L_{I}}$, a measure of the interfacial deflection relative to the depth of the undisturbed interface [20, 48]. Here, $g^{\prime } = \frac{\rho _{2} - \rho _{1}}{\rho _{2}}g$ is the reduced gravity associated with the density difference across the interface, $h_{1}$ is the depth of the epilimnion, $L_{I}$ is the length of the basin at the interface, $u_{*}$ is the wind shear velocity, $\rho _{i}$ is the density of layer $i$, and the subscripts 1 and 2 refer to quantities of the upper and lower layer, respectively. For $W \lesssim 1$, the interface reaches the free surface at the upwind end of the water body, resulting in upwelling of denser fluid into the epilimnion. For continuous profiles with a sharp density interface, partial upwelling is common for $W \lesssim 10$ [28, 29]. For $W \gtrsim 10$, the displacement of the interface is much smaller than the depth of the epilimnion. To calculate $W$, the along-valley component of the wind stress was smoothed using a rolling-average filter with a window size equal to $T_{1} / 4$ [45, 46], where $T_{1}$ is the period of the fundamental internal seiche mode given by $T_{1} = 2L_{I} / \sqrt{g^{\prime }h_{1}h_{2} / \left( h_{1} + h_{2} \right) }$ and $h_{2}$ is the average depth below the epilimnion.

### Density

Given the observed range of conductivity and turbidity, we can neglect the contribution of both to the density of water. Conductivity ranged from 60 to ${100}\,{\upmu \hbox {S}\,\hbox {cm}^{-1}}$ (5th–95th percentile of all CTD profiles), corresponding to a change in density of $\Delta \rho / \rho _{0} \approx 2 \times 10^{- 5}$. Turbidity ranged from $<{1}\,\hbox {NTU}$ to ${30}\,\hbox {NTU}$ (5th–95th percentile), corresponding to order of ${30}\,{\hbox {mg}\,\hbox {L}^{-1}}$ with a change in density of $\Delta \rho / \rho _{0} \approx 2 \times 10^{- 5}$. For comparison, temperature ranged from ${9.6}\,^\circ \hbox {C}$ to ${19.5}\,^\circ \hbox {C}$ (5th–95th percentile), corresponding to a change in density of $\Delta \rho / \rho _{0} \approx 1.4 \times 10^{- 3}$; even for a smaller range of ${10}\,^\circ \hbox {C}$ to ${12}\,^\circ \hbox {C}$ the change in density is $\Delta \rho / \rho _{0} \approx 2 \times 10^{- 4}$. These changes in density due to temperature are one or two orders of magnitude greater than the changes in density due to conductivity or turbidity.

## Results

### Inflows

**Fig. 4:**
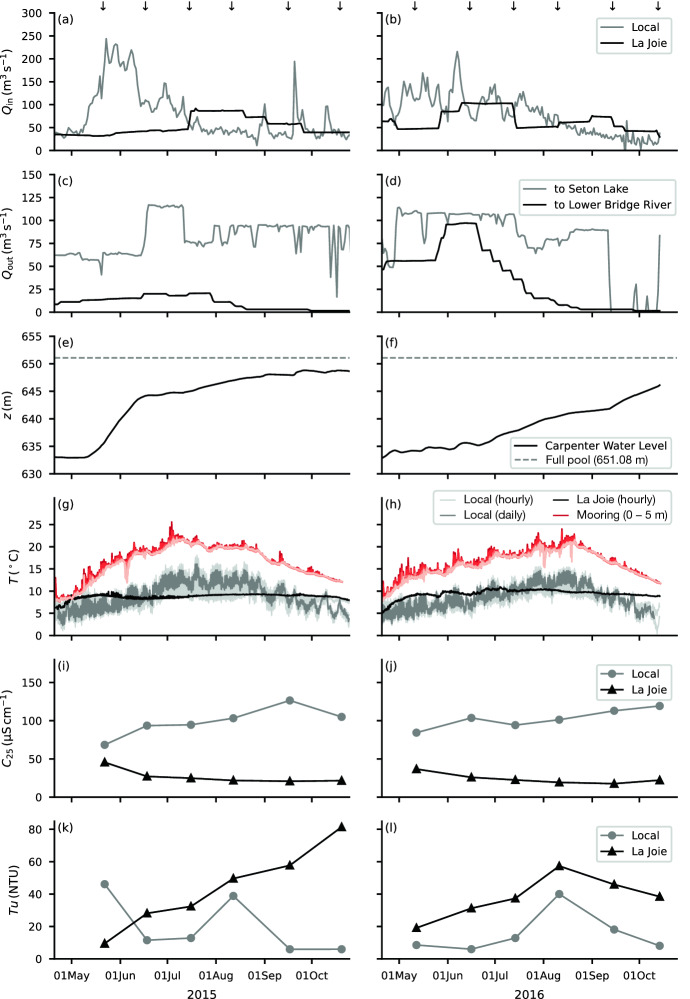
**a, b** Inflows, **c, d** outflows, **e, f** water level, **g, h** tributary temperature, **i, j** conductivity, and **k, l** turbidity in (left) 2015 and (right) 2016. Temperature, conductivity, and turbidity measurements for the local inflow are flow-weighted averages of all the sampled tributaries. The downward arrows mark the time of the reservoir and tributary surveys. In **e, f** the dashed line marks the elevation of full pool (651.08 m asl). In **g, h** the local flow-weighted average tributary temperature is shown hourly (light grey) and daily (grey); the mooring temperature (0–5 m) is shown in shades of red for reference

Inflows into Carpenter Reservoir originate from two main sources: the unregulated inflow from local tributaries, and the regulated inflow from La Joie Dam. In May and June, the inflows are dominated by snowmelt from the local tributaries, while later in the summer they are dominated by inflow from La Joie Dam (Fig. 4a, b). In 2015 there was a large peak in local inflow from late May to early June (Fig. 4a), while in 2016 freshet happened more gradually (Fig. 4b), and in both years the overall local inflow was close to average. Of the outflow, most is diverted through twin tunnels to Seton Lake with the remainder released through twin low-level outlets to the Lower Bridge River (Fig. 4c, d). Both the tunnels and the low-level outlets are deep, drawing water from the bottom of the hypolimnion (Fig. 2c). The water level in the reservoir is drawn down over the winter to generate electricity and typically reaches a minimum in early spring (21 March 2015; 19 April 2016). As the snowpack melts during freshet, the reservoir is allowed to fill (Fig. 4e, f). In 2016, an unusually high volume of water was released to the Lower Bridge River (Fig. 4d), resulting in a water level that was below average for most of the summer (Fig. 4e, f).

### Tributary sampling

The local tributaries were typically warmer than the inflow from La Joie Dam during the warmest part of the summer and cooler for the rest of the year (Fig. 4g, h). While the temperature of the local tributaries followed a seasonal cycle, the temperature from La Joie Dam was relatively steady, being withdrawn from the deep water in Downton Reservoir (Fig. 2a). Both the local and La Joie inflows remained colder than the epilimnion throughout the study period (Fig. 4g, h). The conductivity from La Joie Dam ranged from 20 to ${45}\,{\upmu \hbox {S}\,\hbox {cm}^{-1}}$ (Fig. 4i, j), and that from the local tributaries (flow-weighted average) was also relatively low during freshet (60–100 ${\upmu \hbox {S}\,\hbox {cm}^{-1}}$), though it gradually increased to ${\sim}{120}\,{\upmu \hbox {S}\,\hbox {cm}^{-1}}$ in fall. Turbidity from the local tributaries was lower than that from La Joie Dam, with the only exception being local tributary samples collected on 23 May 2015, the day after a rain event (Fig. 4k, l).

### Wind

**Fig. 5:**
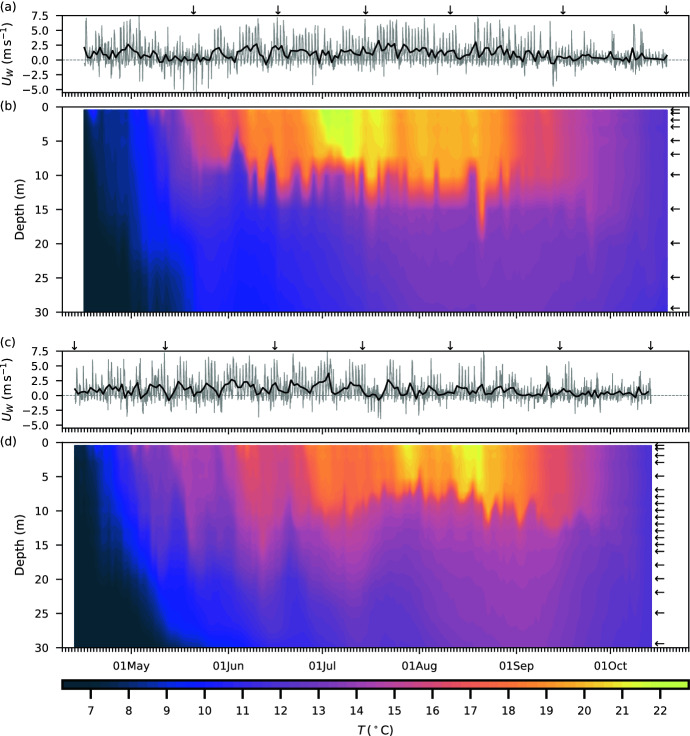
Time series of along-axis wind speed, $U_W$, and contour plots of water temperature, $T$, from 16 April 2015 to 20 October 2015 (**a, b**) and 13 April 2016 to 14 October 2016 (**c, d**). The downward arrows mark the date of the field surveys, and the leftward arrows mark the depth of the temperature sensors at the mooring station near the deep end of the reservoir. **a, c** Positive wind is from the west toward the dam; the grey line marks the hourly wind speed and the black line marks the daily average wind speed

The wind over Carpenter Reservoir was constrained along the valley due to the steep topography bounding the reservoir on the north and south sides (Fig. 2a). The prevailing wind direction was from the west toward the dam (insets, Fig. 2b), consistent with the downslope winds from the Bridge Glacier [40], and characteristic of many mountain glaciers during periods of melting [32]. During the study period the average wind speed was ${2.0}\,{\hbox {m}\,\hbox {s}^{-1}}$; however, the wind showed a strong diurnal pattern, rising in late morning, peaking in the afternoon, and declining to $<{1}\,{\hbox {m}\,\hbox {s}^{-1}}$ in the evening (Fig. 5a, c). The diurnal winds were strongest in June and July when the mean of the daily maximum peaked at ${5.1}\,{\hbox {m}\,\hbox {s}^{-1}}$ and weakest in October when it reached only ${2.8}\,{\hbox {m}\,\hbox {s}^{-1}}$.

### Temperature mooring

Time-series measurements from the temperature mooring are shown as contour plots for 2015 (Fig. 5b) and 2016 (Fig. 5d). At the beginning of the mooring period, on 16 April 2015, the reservoir was slightly stratified with temperature ranging from ${7.4}\,^\circ \hbox {C}$ at the surface to ${5.5}\,^\circ \hbox {C}$ at depth. Temperature stratification was highest during a period of prolonged hot weather, 26 June to 10 July 2015, during which time the epilimnion temperature was above ${20}\,^\circ \hbox {C}$ and reached a maximum of ${24.9}\,^\circ \hbox {C}$ at ${0.5}\,{\hbox {m}}$ during a period of low wind on 3 July 2015 (Fig. 5a). The hypolimnion also warmed over the summer, from ${\sim}{10}\,^\circ \hbox {C}$ in May and reaching ${\sim}{13}\,^\circ \hbox {C}$ in late August 2015, being influenced by the temperature of the inflows. By the final day of the mooring period, on 20 October 2015, little stratification remained with temperature ranging from ${12.2}\,^\circ \hbox {C}$ to ${11.3}\,^\circ \hbox {C}$. Note that in 2015, the internal motions show up as steps because of the limited vertical resolution; in 2016, additional temperature sensors were added to the mooring to better resolve the thermocline.

On 13 April 2016, at the start of the mooring period, the reservoir had just begun to stratify with temperature ranging from ${7.3}\,^\circ \hbox {C}$ to ${4.6}\,^\circ \hbox {C}$. Unlike in 2015, when the reservoir reached maximum temperature stratification from late June to early July, in 2016 the maximum stratification occurred from late July to early August, with temperature at ${0.5}\,{\hbox {m}}$ reaching ${22.9}\,^\circ \hbox {C}$ on 28 July 2016 and ${23.2}\,^\circ \hbox {C}$ on 12 August 2016 during periods of hot weather and low wind (Fig. 5c, d). Over the summer, the temperature of the hypolimnion again warmed, with temperature at ${30}\,\hbox{m}$ reaching a maximum of ${13.6}\,^\circ \hbox {C}$ in early September. By mid-October, little stratification remained with temperature ranging from ${11.9}\,^\circ \hbox {C}$ to ${11.5}\,^\circ \hbox {C}$ on 14 October 2016 when the mooring was recovered. Note the mooring is near the dam; when the daily average wind increases, downwelling of the thermocline is observed.

### Seasons

**Fig. 6:**
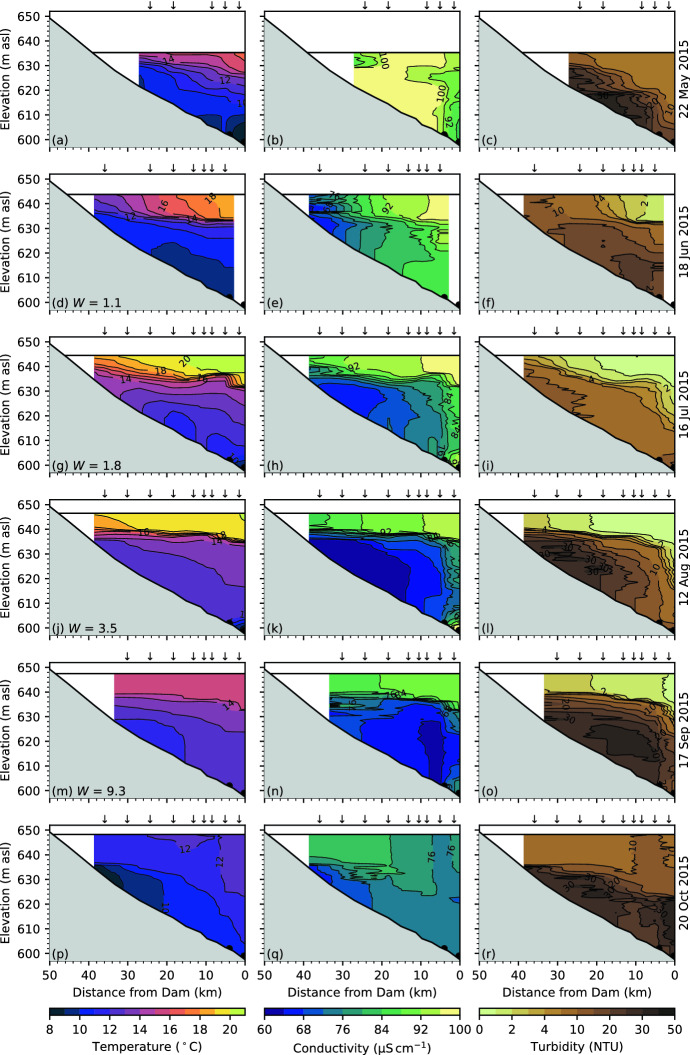
Temperature, conductivity and turbidity in Carpenter Reservoir, May to October 2015. The downward arrows mark the location of the CTD profiles. The black dots mark the tunnel and dam outlets. In **d, g, j, m** the Wedderburn number, $W$, is indicated

**Fig. 7:**
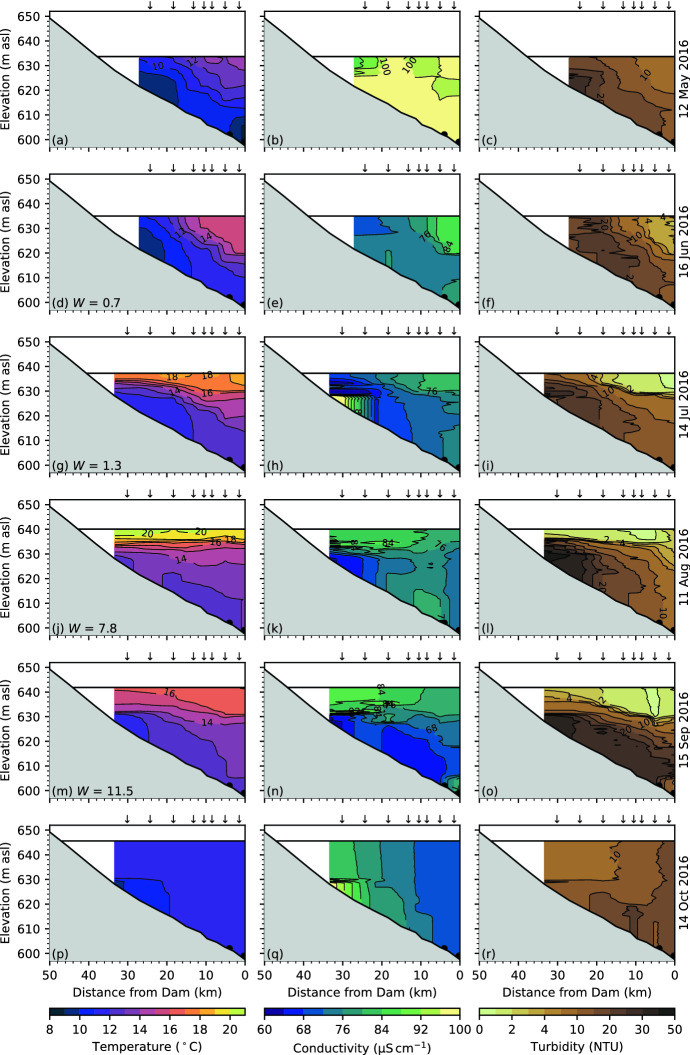
Temperature, conductivity and turbidity in Carpenter Reservoir, May to October 2016. The downward arrows mark the location of the CTD profiles. The black dots mark the tunnel and dam outlets. In **d, g, j, m** the Wedderburn number, $W$, is indicated

While Carpenter Reservoir began to show thermal stratification as early as April, significant vertical exchanges continued to occur for up to three months. For Carpenter Reservoir we define the onset of permanent summer stratification as the first day when the maximum temperature gradient exceeded ${1}\,^{\circ }\hbox {C}\,\hbox {m}^{-1}$ for 90% of the day. In 2015, permanent summer stratification began on 29 May, while in 2016 it did not occur until 14 July. As the timing of permanent summer stratification varied from year to year, we define the seasons accordingly. The period from ice-off to the onset of permanent summer stratification is defined as spring, from the onset of permanent summer stratification to the onset of fall deepening in late August as summer, and from the onset of fall deepening to ice-on as fall. In what follows, we first focus on the data collected in 2015, looking at the profile data shown as contour plots in Fig. 6. Then we compare these with profile data collected in 2016, shown in Fig. 7.

#### Spring 2015

The first survey, on 22 May 2015, occurred near the peak of freshet when the tributary inflow reached ${244}\,{\hbox {m}^3\,\hbox {s}^{-1}}$ (23 May 2015, Fig. 4a). At this time, the reservoir was beginning to stratify with temperature ranging from ${15}\,^\circ \hbox {C}$ near the surface to ${8}\,^\circ \hbox {C}$ near the bottom (Fig. 6a). Conductivity was relatively high and uniform (${\sim }{100}\,{\upmu \hbox {S}\,\hbox {cm}^{-1}}$, Fig. 6b), and provides a useful tracer of water masses in the reservoir. Turbidity was highest in the deep water due to plunging of cold and turbid inflows below the warmer surface layer; these inflows then travelled through the hypolimnion to the deep outlets (Fig. 6c).

#### Summer 2015

From the survey of 22 May to that of 18 June 2015, inflow from the local tributaries declined by more than half to ${107}\,{\hbox {m}^3\,\hbox {s}^{-1}}$, though this was still higher than the inflow from La Joie Dam (Fig. 4a). Near the dam, the epilimnion had warmed to ${18}\,^\circ \hbox {C}$ and a sharp thermocline developed at ${\sim\!\!10}\,{\hbox {m}}$ depth (Fig. 6d); however, along most of the reservoir, isotherms intersected the surface, likely the result of wind-driven upwelling. A similar pattern was observed in the conductivity and turbidity of the epilimnion (Fig. 6e, f). From 22 May to 18 June 2015, the epilimnion freshened only slightly (Fig. 6e), indicating that thermal stratification kept the epilimnion relatively isolated from the lower-conductivity inflows (Fig. 4i). In contrast, a significant decline in conductivity occurred during this time in the hypolimnion from ${\sim}{100}\,{\upmu \hbox {S}\,\hbox {cm}^{-1}}$ to 70–90 ${\upmu \hbox {S}\,\hbox {cm}^{-1}}$ (Fig. 6e), reflecting the lower conductivity of the plunging inflows. From May to June 2015, the turbidity near the surface declined along the length of the reservoir, dropping the most near the dam from just above ${10}\,\hbox {NTU}$ to less than ${2}\,\hbox {NTU}$ (Fig. 6c, f).

At the time of the field survey on 16 July 2015, the reservoir showed a sharp thermocline near the dam. In addition, there were signs of internal motions along the thermocline and upwelling at the upstream end (Fig. 6g). The epilimnion retained the relatively high conductivity observed in May and June (Fig. 6h), and the turbidity in the epilimnion continued to decline to ${\sim}{1}\,\hbox {NTU}$ (Fig. 6i). This is in contrast to the hypolimnion, where the conductivity continued to decline and the turbidity remained high and variable, reflecting the changing composition of the inflows over the summer.

From 16 July to 12 August 2015, the thermal stratification remained much the same, with temperature ranging from ${19}\,^\circ \hbox {C}$ in the epilimnion to ${13}\,^\circ \hbox {C}$ in the hypolimnion, and a sharp thermocline at ${7}\,{\hbox {m}}$ depth (Fig. 6j). In the epilimnion, conductivity remained relatively constant, having declined only slightly from July (Fig. 6k), and turbidity remained low ($<{0.8}\,\hbox {NTU}$, Fig. 6l). In contrast, in the hypolimnion conductivity continued to decline to 75–65 ${\upmu \hbox {S}\,\hbox {cm}^{-1}}$, accompanied by an increase in turbidity to 10–20 NTU (Fig. 6k, l), reflecting the increasing proportion of fresh and turbid inflow from La Joie Dam (66% of the total inflow, Fig. 4a).

#### Fall 2015

By 17 September 2015, the epilimnion had begun to cool and had deepened slightly to ${\sim}{12}\,{\hbox {m}}$ (Fig. 6m). As the surface layer deepened, fresher and more turbid water from below was entrained into the epilimnion, reducing the conductivity of the epilimnion to ${\sim}{90}\,{\upmu \hbox {S}\,\hbox {cm}^{-1}}$ (Fig. 6n), and increasing the turbidity to ${\sim}{2}\,\hbox {NTU}$ (Fig. 6o). The survey on 20 October 2015 occurred just before fall turnover, and the epilimnion had deepened significantly, reaching ${\sim}{25}\,{\hbox {m}}$ near the dam (Fig. 6p). By this time, the conductivity of the epilimnion had declined to ${\sim}{75}\,{\upmu \hbox {S}\,\hbox {cm}^{-1}}$, considerably less than at the beginning of the field season, and little contrast in conductivity remained with the deep water (Fig. 6q). As the surface layer deepened, the turbidity of the epilimnion rose to ${\sim}{10}\,\hbox {NTU}$, a result of mixing with water from the hypolimnion where turbidity remained high (20–35 NTU, Fig. 6r).

#### 2016 field season

Overall, conditions in 2016 were similar to those in 2015 with a few notable exceptions. As described earlier, the water level began low and rose more slowly than in 2015 (Fig. 4e, f) and higher outflows occurred in June 2016 (Fig. 4d). The high outflows were the result of a decision by the hydroelectric utility to lower the water level in Downton Reservoir (Fig. 2a), upstream of Carpenter Reservoir, to mitigate the seismic risk of the ageing dam. The lowering of the water level in Downton Reservoir resulted in abnormally high inflows into Carpenter Reservoir, which, in turn, led to high outflows to the Lower Bridge River and to Seton Lake (Fig. 4d). The low water level and high flushing rates may have contributed to a delay in permanent summer stratification in 2016, which was not observed in the June survey (Fig. 7d), though it had been established by the July survey (Fig. 7g).

### Upwelling and interfacial displacements

The prevailing westerlies result in the potential for upwelling at the upstream end of Carpenter Reservoir. The internal seiche period is ${\sim}{4}$ days through much of the summer and the average wind stress over the one-quarter seiche period (${\sim}{1}$ day) sets the magnitude of upwelling (Sect. 2.4). For example, on 18 June 2015 the strong sustained down-valley winds led to a deeper thermocline near the dam than at the upstream end of the reservoir (Fig. 6d). Isotherms at the top of the metalimnion were brought to the surface at the upwind end of the reservoir, introducing colder metalimnetic fluid to the epilimnion and, in turn, setting up a longitudinal gradient in temperature (Fig. 6d). At the time of the next field survey on 16 July 2015, the strong diurnal winds again resulted in an overall tilting of the thermocline toward the dam as well as generating what appear to be non-linear internal wave motions near the dam (Fig. 6g). In contrast, during light winds on 12 August 2015, the thermocline was nearly horizontal with only a slight deepening near the dam and only a slight lifting of the ${19}\,^\circ \hbox {C}$ isotherm at the upstream end of the reservoir. For comparison, in 2016, the highest potential for upwelling occurred on 16 June 2016 when the Wedderburn number fell below one ($W = 0.7$, Fig. 7d).

### Transport of plunging inflows through the hypolimnion

While this paper focuses on the epilimnion, both the low residence time of the hypolimnion and the high flux of turbidity through the hypolimnion warrant additional comment. We define the bulk residence time of the hypolimnion, $\tau _{\mathrm {hypo}} = V_{\mathrm {hypo}} / Q_{\mathrm {out}}$, where $Q_{\mathrm {out}}$ is the outflow from the reservoir, and $V_{\mathrm {hypo}}$ is the volume of the hypolimnion. From mid-June to mid-September, $\tau _{\mathrm {hypo}}$ was on average 60 days in 2015 and only 40 days in 2016 when outflows were higher (Fig. 4c, d). It was even shorter from 30 May to 18 June 2016, a period of low water level (Fig. 4f) and high outflow (Fig. 4d), when $\tau _{\mathrm {hypo}}$ fell to 15 days. Note that $\tau _{\mathrm {hypo}}$ gives an upper bound on the residence time because stratification inhibits uniform mixing throughout $V_{\mathrm {hypo}}$, for example, when inflows travelled as a gravity current along the bottom of the reservoir as indicated by the data in Figs. 6g–i and 7g–i.

The low residence time of the hypolimnion had important consequences for the reservoir and for the outflow. First, the temperature of the hypolimnion (Fig. 5b, d), and of the outflow, rose over the course of the summer, tracking the mean temperature of the inflows. For example, by the end of August the average temperature of the hypolimnion at the mooring had reached ${13.0}\,^\circ \hbox {C}$ in 2015 and ${13.6}\,^\circ \hbox {C}$ in 2016. Note that the rising temperature of the hypolimnion reduced the temperature difference across the thermocline. Second, the short residence time left little opportunity for settling of glacial fines from the hypolimnion. This is confirmed by the available measurements of tributary turbidity: the flux of turbidity entering the hypolimnion with the inflows was roughly balanced by that in the outflow to Seton Lake and the Lower Bridge River. Note that the flux of turbidity entering the hypolimnion from the inflows was an order of magnitude higher than the flux that settled out from the epilimnion. The remarkably short residence time, coupled with the through flow of temperature and turbidity, suggests that the hypolimnion in Carpenter Reservoir was not a hypolimnion in the traditional sense, namely, one in which the temperature is set by spring turnover and changes little over the summer [50].

### Seasonal variation of epilimnetic turbidity

**Fig. 8:**
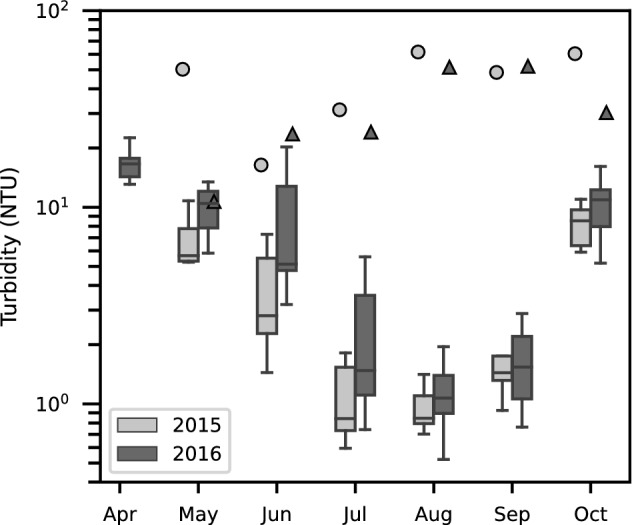
Boxplot showing turbidity at ${1}\,{\hbox {m}}$ depth at stations C1–C9. The markers indicate turbidity measurements collected at Middle Bridge below Hurley River (the inflow from La Joie Dam plus Hurley River) in 2015 (circles) and 2016 (triangles)

The turbidity in the epilimnion varied seasonally, with high turbidity in the spring, decreasing during the summer, and increasing again in the fall (Fig. 8). In May 2015, the epilimnetic turbidity was high, ranging from 5 to 10 NTU. Permanent summer stratification began on 29 May 2015 after which time the epilimnion became relatively isolated from potential sources of higher turbidity from the hypolimnion. From June to August 2015, the turbidity followed a characteristic exponential decay [37, 42] with an e-folding time of ${\sim}{40}$ days (2.5% decrease per day). In September and October, the epilimnion deepened, which mixed in turbid water from below, increasing the epilimnetic turbidity to ${\sim}{10}\,\hbox{NTU}$ by October.

In 2016, the turbidity was similar. The additional survey in April 2016 yielded the highest turbidity (15–20 NTU), decreasing by about one half in May (5–10 NTU), similar to the turbidity in May 2015. In June 2016, the turbidity was generally higher than in 2015 (Fig. 8); permanent summer stratification did not occur until after the survey in June. During the summer of 2016 (July and August) turbidity declined with an e-folding time of ${\sim}{30}$ days (3.3% decrease per day). From August to October, the turbidity followed a similar pattern as in 2015.

Tedford et al. [47] observed similar seasonal variation in turbidity, from spring to fall, in their study of Base Mine Lake. This seasonal pattern is likely to occur in other turbid lakes with high concentrations of fine particulate matter.

### Longitudinal variation of epilimnetic turbidity

**Fig. 9:**
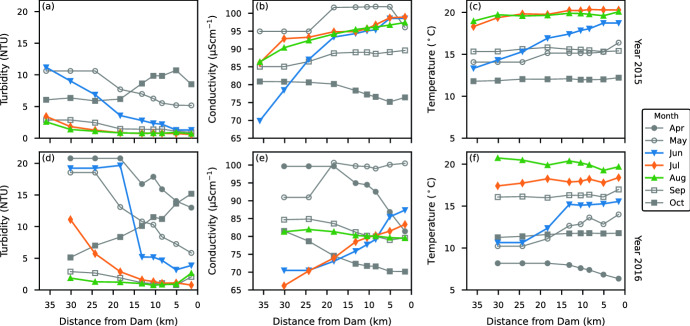
Variation of turbidity (**a, d**), conductivity (**b, e**), and temperature (**c, f**) in the epilimnion at the stations along the length of Carpenter Reservoir in 2015 (C1–C9; **a, b, c**) and 2016 (C1–C8; **d, e, f**)

From June to August, turbidity in the epilimnion was consistently highest at the upstream end of the reservoir nearest to the plunging glacial inflow, and lowest at the downstream end near the dam. Also observed were consistent longitudinal gradients in the temperature and conductivity of the epilimnion, with the coolest and freshest water at the upstream end of the reservoir. These longitudinal gradients persisted throughout the summer (Fig. 9).

Consider for example, June 2015, following the onset of permanent summer stratification: the turbidity at the upstream end remained elevated (${\sim}{12}\,\hbox{NTU}$), while the turbidity near the dam was already ${\sim}{1.5}\,\hbox{NTU}$. The elevated turbidity at the upstream end of the reservoir can be explained by the down-valley winds observed during the June survey, resulting in the upwelling of cooler, more turbid metalimnetic fluid into the epilimnion ($W = 1.1$, Fig. 6d). By July 2015, the turbidity had declined to 4 NTU at the upstream end, and to 1 NTU at the dam (Fig. 9a).

## Theoretical analysis of longitudinal turbidity variation in the epilimnion

Our measurements reveal that the epilimnion of Carpenter Reservoir is surprisingly isolated from the cold glacial inflows, which pass through the hypolimnion to the deep outlets. While the turbidity in the epilimnion is high in spring, it decreases throughout summer, and only begins to increase again as the epilimnion deepens in fall. However, despite the relative isolation of the epilimnion, the data suggest that a small fraction of inflow is transported into the epilimnion. Here we look at a theoretical analysis of the changes in turbidity of the epilimnion during summer.

To estimate the variation of turbidity in the epilimnion during summer, we derive an analytical model that incorporates longitudinal dispersion and particle settling. Since we use turbidity as a proxy for light attenuation, the model will help us characterize the light regime in Carpenter Reservoir, one of our primary goals. Since there is no outlet from the epilimnion we neglect advection. The variation of turbidity in the epilimnion can then be described by the one-dimensional (longitudinal) diffusion equation for a decaying substance, which can be written as

1 $$\begin{aligned} \frac{\partial C_*}{\partial t_*} = K \frac{\partial ^2 C_*}{\partial x_*^2} - \frac{C_*}{\tau _s}, \end{aligned}$$

where $C_{*}$ is concentration (turbidity), $t_{*}$ is time, $x_{*}$ is the distance upstream of the dam, $K$ is the longitudinal dispersion coefficient, and $\tau _{s} = h_{1} / v_{s}$ is the particle settling time scale, where $h_{1}$ is the depth of the epilimnion and $v_{s}$ is the particle settling velocity. Equation (1) can be rewritten in nondimensional form by scaling the dimensional variables (identified with asterisks) by the following length, time, and concentration scales:

2 $$\begin{aligned} x = \frac{x_*}{L}, \quad t = \frac{t_*}{\tau _s}, \quad C = \frac{C_*}{\bar{C}_{0_*}}, \end{aligned}$$

where $L$ is the distance from the dam to the location of the most upstream survey (C9 in 2015 and C8 in 2016), and ${\bar{C}}_{0_{*}} = 1 / L\int _{0}^{L}{C_{*}\left( x_{*},t_{0_{*}} \right) \mathrm {d}x_{*}}$ is the longitudinal average concentration in the epilimnion at the initial time $t_{*} = t_{0_{*}}$. Substituting (2) into (1) gives

3 $$\begin{aligned} \frac{\partial C}{\partial t} = {\mathcal {D}}\frac{\partial ^2 C}{\partial x^2} - C, \end{aligned}$$

where ${\mathcal {D}}= \frac{Kh_{1}}{L^{2}v_{s}}$. The nondimensional parameter ${\mathcal {D}}$ is the ratio of the particle settling time scale to the dispersive time scale, $\tau _{D} = \frac{L^{2}}{K}$. The inverse of this parameter is the limnological equivalent of the Damköhler number used in chemical engineering to relate a transport time scale (advective or diffusive) to a chemical reaction time scale [7, 33].

The variation of turbidity is determined by solving (3) subject to the following initial, final, and boundary conditions:

4a $$\begin{aligned} C(x,t_0) = C_0(x),\quad C(x,t_f) = C_f(x), \end{aligned}$$

4b $$\begin{aligned} \frac{\partial C}{\partial x}(0, t) = 0, \quad \frac{\partial C}{\partial x}(1, t) = \frac{\mathcal {I}}{{\mathcal {D}}}. \end{aligned}$$

In (4a), $C_{0}\left( x \right)$ is the measured variation of turbidity along the length of the reservoir at the initial time $t = t_{0}$, $C_{f}\left( x \right)$ is the measured variation of turbidity along the length of the reservoir at the final time $t = t_{f}$. Equation (4b) imposes a no flux boundary condition at the dam wall and a constant flux boundary condition at the upstream end of the epilimnion, where $\mathcal {I}= {\dot{M}}_{\mathrm {epi}} / {\dot{M}}_{s}$. The nondimensional parameter $\mathcal {I}$ is the ratio of the mass flow rate into the upstream end of the epilimnion, ${\dot{M}}_{\mathrm {epi}}$, to the mass flow rate out of the epilimnion due to particle settling, ${\dot{M}}_{s}$, or, equivalently, the ratio of the particle settling time scale to the particle influx time scale, $\tau _{I} = \frac{{\overline{C}}_{0_{*}}V_{\mathrm {epi}}}{{\dot{M}}_{\mathrm {epi}}}$, where $V_{\mathrm {epi}}$ is the volume of the epilimnion.

Note in (2), $t_{*}$ is nondimensionalized by $\tau _{s}$, which is unknown *a priori*; therefore, the nondimensional duration of the summer, $\mathcal {T}= t_{f} - t_{0}$, is also an unknown. As a result, (3) depends on three nondimensional parameters: $\mathcal {I}$, ${\mathcal {D}}$, and $\mathcal {T}$. To simplify the analysis, we consider the longitudinal average of (3), which is given by

5 $$\begin{aligned} \frac{\mathrm {d}\bar{C}(t)}{\mathrm {d}t}= & {} \mathcal {I}- \bar{C}(t), \nonumber \\ \bar{C}\left( t_0 \right)= & {} 1, \end{aligned}$$

where $\bar{C}\left( t \right)$ is the longitudinal average nondimensional turbidity. The solution to (5) can be written as

6 $$\begin{aligned} \bar{C}\left( t \right) = 1 + \left( \mathcal {I}- 1 \right) \left( 1 - e^{- \left( t - t_{0} \right) } \right) . \end{aligned}$$

Evaluating (6) at $\ t = t_{f}$, yields

7 $$\begin{aligned} \mathcal {I}= \frac{\bar{C}\left( t_{f} \right) - e^{- \mathcal {T}}}{1 - e^{- \mathcal {T}}}, \end{aligned}$$

leaving $\mathcal {I}$ and ${\mathcal {D}}$ as our unknowns. Note that since $\mathcal {I}$ is the ratio of the particle settling time scale to the particle influx time scale,

8 $$\begin{aligned} \mathrm {if} \;\; {\left\{ \begin{array}{ll} \mathcal {I}< 1,\quad \text {then}\;\tau _s < \tau _I,\;\text {particle settling exceeds particle influx and}\;\bar{C}(t)\;\text {decreases with}\;{t.}\\ \mathcal {I}= 1,\quad \text {then}\;\tau _s = \tau _I\; \text {and}\; \bar{C}(t)\;\text {remains constant.} \\ \mathcal {I}> 1,\quad \text {then}\;\tau _s > \tau _I,\;\text {particle influx exceeds particle settling and}\;\bar{C}(t)\;\text {increases with}\;{t.} \end{array}\right. } \end{aligned}$$

A consequence of (8) is that $\mathcal {I}$ can serve as a bound on $\bar{C}\left( t \right)$—a lower bound if $\mathcal {I}< 1$ and an upper bound if $\mathcal {I}> 1$.

After a long enough time, the longitudinal variation of turbidity described by (3) approaches a steady state where longitudinal dispersion is balanced by particle settling. The steady-state solution is

9 $$\begin{aligned} C_{\infty }(x) = \frac{\mathcal {I}}{\sqrt{{\mathcal {D}}}} \frac{\cosh {\left( \frac{x}{\sqrt{{\mathcal {D}}}}\right) }}{\sinh {\left( \frac{1}{\sqrt{{\mathcal {D}}}}\right) }}. \end{aligned}$$

**Fig. 10:**
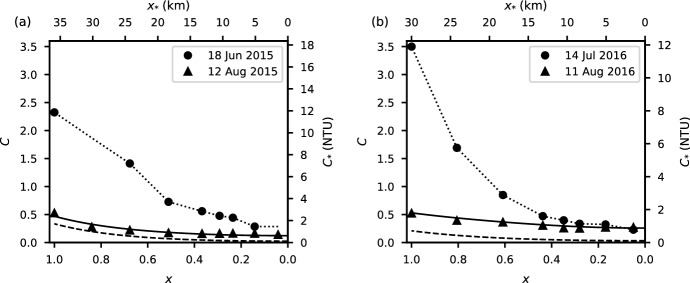
Variation of turbidity in the epilimnion with distance from the dam in 2015 (**a**) and 2016 (**b**). The solid circles and triangles are field measurements; the dotted lines are the initial conditions linearly interpolated from the field measurements; the solid lines are the best fit solutions to (3) at the final time $t=t_f$. The dashed lines are the steady-state solutions to (9). The dam is at $x=0$

If $t$ is sufficiently large, then the curve $C_{\infty }\left( x \right)$ provides a useful bound on $C\left( x,t \right)$, see Fig. 10a, b. Also, if (9) is integrated over $x$, then ${\overline{C}}_{\infty } = \mathcal {I}$, which is equivalent to the limit of (6) as $t \rightarrow \infty$.

Four regimes of epilimnetic turbidity variation (temporal and longitudinal) during summer can be identified based on the values of the epilimnetic inflow parameter, $\mathcal {I}$, and the dispersion parameter, ${\mathcal {D}}$:

- Regime I: ($\mathcal {I}< 1$, ${\mathcal {D}}< 1$) turbidity decreasing with *t* with a strong longitudinal gradient,
- Regime II: ($\mathcal {I}> 1$, ${\mathcal {D}}< 1$) turbidity increasing with *t* with a strong longitudinal gradient,
- Regime III: ($\mathcal {I}< 1$, ${\mathcal {D}}> 1$) turbidity decreasing with *t* with a weak longitudinal gradient,
- Regime IV: ($\mathcal {I}> 1$, ${\mathcal {D}}> 1$) turbidity increasing with *t* with a weak longitudinal gradient.

## Discussion

Here we apply the theory presented in Sect. 4 to estimate the particle settling velocity, longitudinal dispersion coefficient, and flux of turbid water into the epilimnion at the upstream end of Carpenter Reservoir. Then we discuss conditions under which the turbidity in the epilimnion could significantly change.

### Application of the theoretical analysis to Carpenter Reservoir

**Table 1:** Definitions and values of governing parameters

Expression	Description	Summer 2015$^\mathrm{a}$	Summer 2016$^\mathrm{a}$	Units
*Governing parameters*				
${\mathcal {D}}=\frac{K h_1}{L^2 v_s} = \frac{\tau _s}{\tau _D}$	Dispersion parameter	0.09	0.15	–
$\mathcal {I}=\frac{\dot{M}_{\mathrm {epi}}}{\dot{M}_{\mathrm {s}}} = \frac{\tau _s}{\tau _I}$	Epilimnetic inflow parameter	0.10	0.08	–
$\mathcal {T} = t_f - t_0$	Nondimensional time over summer	2.0	1.4	–
*Time scales*				
$\tau _D = \frac{L^2}{K}$	Dispersive time scale	290	150	d
$\tau _I = \frac{\bar{C}_{0_*}V_{\mathrm {epi}}}{\dot{M}_{\mathrm {epi}}}$	Particle influx time scale	260	290	d
$\tau _s = \frac{h_1}{v_s}$	Particle settling time scale	26	23	d
*Measured quantities*				
*L*	Length of the reservoir$^\mathrm{b}$	36	30	km
$h_1$	Epilimnion depth	7	5	m
$V_{\mathrm {epi}}$	Epilimnion volume	250	150	$\mathrm {Mm}^3$
$\bar{C}_{0_*}$	Initial turbidity of the epilimnion$^\mathrm{c}$	5.1	3.4	NTU
$\bar{C}_{f_*}$	Final turbidity of the epilimnion$^\mathrm{c}$	1.1	1.3	NTU
*Model-derived parameters*				
$v_s$	Stokes particle settling velocity	0.27	0.22	$\mathrm{m} \, \mathrm {d}^{-1}$
*K*	Longitudinal dispersion coefficient	52	68	$\mathrm{m}^{2} \, \mathrm {s}^{-1}$
$\dot{M}_{\mathrm {epi}}$	Mass flow rate into the epilimnion$^\mathrm{d}$	57	20	$\mathrm{m}^{3} \, \mathrm{s}^{-1} \, \mathrm{NTU}$
*Turbidity inflow*				
$\alpha =\frac{\dot{M}_{\mathrm {epi}}}{\dot{M}_{\mathrm {in}}}$	Turbidity inflow ratio	0.015	0.005	–
$\dot{M}_{\mathrm {in}} = Q_{\mathrm {in}} C_{\mathrm {in}}$	Mass flow rate into the reservoir	3700	4100	$\mathrm{m}^{3} \, \mathrm{s}^{-1} \, \mathrm{NTU}$
$\dot{M}_{\mathrm {s}} = Q_{\mathrm {s}}\bar{C}_{0_*}$	Mass flow rate of particle settling$^\mathrm{e}$	570	250	$\mathrm {m}^{3} \, \mathrm{s}^{-1} \, \mathrm{NTU}$
$Q_{\mathrm {in}}$	Volumetric flow rate of inflow$^\mathrm{f}$	100	120	$\mathrm{m}^{3} \, \mathrm{s}^{-1}$
$C_{\mathrm {in}}$	Turbidity of inflow into the reservoir$^\mathrm{g}$	37	34	NTU
$Q_{\mathrm {s}} = \frac{v_s V_{\mathrm {epi}}}{h_1}$	Volumetric flow rate of settling$^\mathrm{e}$	110	75	$\mathrm {m}^{3} \, \mathrm{s}^{-1}$

$^\mathrm{a}$Summer 2015: 18 June to 12 August; Summer 2016: 14 July to 11 August

$^\mathrm{b}$Defined as the distance from the dam to the location of the most upstream survey

$^\mathrm{c}$Defined as the longitudinal average concentration

$^\mathrm{d}$Flux into the epilimnion at the upstream end of the reservoir

$^\mathrm{e}$Particle settling out of the epilimnion

$^\mathrm{f}$Flow from La Joie Dam, Hurley River, and Gun Creek time averaged over the summer

$^\mathrm{g}$Flow-weighted average turbidity time averaged over the summer

To estimate the values $\mathcal {I}$ and ${\mathcal {D}}$ giving the best fit to the field data, (3) was solved numerically for a range of values of $\mathcal {I}$ from 0 to $\overline{C}(t_{f})$, and ${\mathcal {D}}$ from 0 to 1. Field data from the first survey of the summer (18 June in 2015 and 14 July in 2016) were used as the initial condition (circles, Fig. 10a, b). The model was stepped forward in time until the date of the final survey of the summer (12 August 2015 and 11 August 2016, triangles, Fig. 10a, b). Field data from the final survey were compared with the solution to (3), and the values of $\mathcal {I}$ and ${\mathcal {D}}$ yielding the minimum root mean square error were deemed the best fit (solid line, Fig. 10a, b, Table 1).

The method described above provides a means to estimate $\mathcal {I}$, ${\mathcal {D}}$, and $\mathcal {T}$ from which $v_{s}$, $K$, and ${\dot{M}}_{\mathrm {epi}}$ are readily obtained (Table 1). Here we apply this method to the turbidity data collected in the epilimnion of Carpenter Reservoir and discuss our estimates of the latter three parameters. The analysis gives a particle settling velocity of ${0.27}\,{\hbox {m}\,\hbox {d}^{-1}}$ in 2015 with a similar value of ${0.22}\,{\hbox {m}\,\hbox {d}^{-1}}$ in 2016 (Table 1). Assuming Stokes’ law, these estimates correspond to an effective particle diameter of ${\sim}{2}\,\upmu \hbox {m}$, which is similar to values reported in nearby glacier-fed lakes, such as Lillooet Lake, $d_{50} \approx {1.3}\,\upmu \hbox {m}$ [17] and Chilko Lake (Tŝilhqox Biny), $d_{50} \approx$ 4 to ${6}\,\upmu \hbox {m}$ [8]. Our estimates are also consistent with the size range for glacial flour, from 0.7 to ${4}\,\upmu \hbox {m}$, reported for a number of alpine and arctic lakes as collated by Eder [9, Table 10].

Our analysis yields a longitudinal dispersion coefficient in the epilimnion of Carpenter Reservoir of ${52}\,{\hbox {m}^{2}\,\hbox {s}^{-1}}$ in 2015 and ${68}\,{\hbox {m}^{2}\,\hbox {s}^{-1}}$ in 2016 (Table 1). These estimates are consistent with values from 30 to ${100}\,{\hbox {m}^{2}\,\hbox {s}^{-1}}$ obtained from dye tracer experiments in wide ($>{60}\,{\hbox {m}}$), slow-moving ($<{0.4}\,{\hbox {m}\,\hbox {s}^{-1}}$) rivers reported in Rutherford [39, Table 4.2].

Our analysis also shows that the proportion of the turbidity flux entering the reservoir that was transported into the epilimnion, $\alpha = {\dot{M}}_{\mathrm {epi}}\mathrm {/}{\dot{M}}_{\mathrm {in}}$, was 0.015 and 0.005 in 2015 and 2016, respectively (Table 1). These low values are consistent with the fact that during the summers of 2015 and 2016 the inflowing tributaries were all cooler than the epilimnion (Fig. 4g, h), and formed a turbid density current that remained largely intact as it flowed under the epilimnion. We hypothesize that processes such as mixing in the plunge zone [6], internal wave motions [13], peeling detrainment [18], and wind-driven upwelling [28] were responsible for the small proportion of the turbidity flux (${\sim}{1}{\%}$) that made its way into the epilimnion. Note that the wind event on 16 June 2016 with $W = 0.7$ (Fig. 7d) occurred before the start of the summer periods modelled here. During these summer periods $W > 1$ except for a few brief periods, so we expect that wind-driven upwelling events in summer are infrequent.

In both 2015 and 2016 the values of $\mathcal {I}$ and ${\mathcal {D}}$ were similar and of $\mathcal {O}\left( 0.1 \right)$, see Table 1. These low values mean that the summer epilimnetic turbidity in Carpenter Reservoir fell clearly into Regime I (epilimnetic turbidity decreasing with time with a strong longitudinal gradient), as is apparent in Fig. 10. The initial upstream turbidity levels were high, 11.8 NTU in June 2015 and 12.1 NTU in July 2016. However, they dropped rapidly in space and time, and at the end of the summer the average epilimnetic turbidity was only 1.1 NTU in 2015 and 1.3 NTU in 2016.

**Fig. 11:**
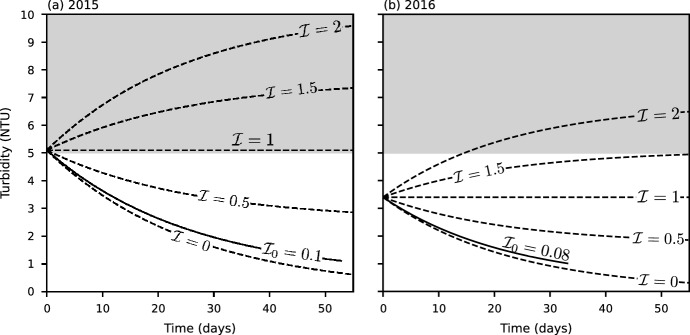
Longitudinal average turbidity, $\bar{C}(t)$, as a function of time for $\mathcal {I}=0,\;0.5,\;1,\;1.5 \; \text {and} \; 2$ (dashed lines); estimated based on field data $\mathcal {I}_0$ (solid line) in 2015 (**a**) and 2016 (**b**). The grey shading indicates that the turbidity is greater than 5 NTU

### Conditions under which the turbidity in the epilimnion could significantly change

From an ecological perspective, elevated turbidity in the epilimnion during summer can be a concern. For example, 5 NTU ($k_{\mathrm {PAR}} = 0.6 \; \mathrm{m}^{-1}$) has been suggested as the threshold for the beginning of light limitation for primary production in glacial lakes [10], and the threshold above which glacial flour can interfere with filter-feeding in cladocerans [24], a key food source for kokanee. In 2015 and 2016, the measured epilimnetic turbidities were only above 5 NTU at the start of the summer and at the two most upstream stations (Fig. 10).

An important question is under what conditions might the average epilimnetic turbidity exceed 5 NTU more often? If the tributaries had been warmer and/or the epilimnion cooler, then $\alpha$, and consequently $\mathcal {I}$, would have been greater. In 2015, for example, if $\alpha$ had increased above 0.16, then $\mathcal {I}>1$, and the reservoir would have been in Regime II. If this had been the case, then the average epilimnetic turbidity would have remained above 5 NTU throughout the summer (Fig. 11a). In 2016, if $\alpha$ had increased to 0.1, corresponding to an increase in $\mathcal {I}$ to 1.6, then the average epilimnetic turbidity would have increased from its initial value of 3.4 NTU to 5 NTU by the end of summer (Fig. 11b).

## Summary and conclusions

Turbidity variations in Carpenter Reservoir were investigated revealing that during summer the vast majority of the turbid inflow plunges deep into the reservoir, passing through the hypolimnion to the deep outlets. Thermal stratification isolates the epilimnion from the turbid inflow and despite the high load of turbidity into the reservoir, the epilimnion clears due to particle settling. The epilimnetic turbidity during summer depends primarily on four factors: the initial turbidity at the onset of permanent summer stratification, the particle settling velocity, the longitudinal dispersion, and the epilimnetic inflow at the upstream end of the reservoir.

A theoretical analysis based on the one-dimensional diffusion equation for a decaying substance is presented. The theory—describing the longitudinal turbidity variation in the epilimnion—depends on two nondimensional parameters: the epilimnetic inflow parameter, $\mathcal {I}$, and the dispersion parameter, ${\mathcal {D}}$. These determine whether the turbidity in the reservoir will increase ($\mathcal {I}> 1$) or decrease ($\mathcal {I}< 1$) and whether the longitudinal gradient in turbidity will be weak (${\mathcal {D}}> 1$) or strong (${\mathcal {D}}< 1$). The theory was applied to turbidity measurements collected in Carpenter Reservoir in 2015 (2016), yielding values of $\mathcal {I}= 0.10\; (0.08)$ and ${\mathcal {D}}= 0.09\; (0.15)$, indicating that the reservoir is in Regime I: turbidity decreasing with time with a strong longitudinal gradient.

For the turbidity data collected in Carpenter Reservoir in 2015 (2016), the analysis led to a value for the particle settling velocity of ${0.27}\,{\hbox {m}\,\hbox {d}^{-1}}$ (${0.22}\,{\hbox {m}\,\hbox {d}^{-1}}$), the longitudinal dispersion coefficient of ${52}\,{\hbox {m}^{2}\,\hbox {s}^{-1}}$ (${68}\,{\hbox {m}^{2}\,\hbox {s}^{-1}}$), and the turbidity inflow ratio of 1.5% (0.5%). The close match for the estimates from both field seasons suggests that a model of the form given by (3) can help identify physical parameters driving variations in epilimnetic turbidity. The estimated parameters also agreed favourably with published data reporting on similar water bodies, providing additional support to the approach.

A common limitation of many field monitoring programs is the time interval between surveys. The theoretical framework presented here is a novel approach for evaluating key physical parameters and is well-suited to datasets consisting of monthly CTD surveys, as in our case.
